# Lymphatic Disorders in Patients With Single Ventricle Heart Disease

**DOI:** 10.3389/fped.2022.828107

**Published:** 2022-06-10

**Authors:** Yoav Dori, Christopher L. Smith

**Affiliations:** Department of Cardiology, Jill and Mark Fishman Center for Lymphatic Disorders, Children’s Hospital of Philadelphia, Philadelphia, PA, United States

**Keywords:** lymphatics, protein-losing enteropathy (PLE), plastic bronchitis, chylothorax, DCMRL, lymphangiography, ascites

## Abstract

Lymphatic abnormalities in patients with single ventricle physiology can lead to early Fontan failure and severe Fontan complications, such as protein-losing enteropathy (PLE), plastic bronchitis (PB), chylothorax, and edema. Recent developments in lymphatic imaging and interventions have shed new light on the lymphatic dysfunction in this patient population and the role of the lymphatic circulation in PLE, PB, and chylothorax. In this study, we reviewed some of the latest developments in this field and discuss new treatment options for these patients.

## Introduction

The lymphatic system plays a key role and has three main functions, namely, immune regulation, long-chain fatty acid absorption, and tissue fluid circulation. Several decades ago, the lymphatic system was studied extensively. However, due to a lack of a simple lymphatic imaging technique and lymphatic interventional techniques, the system was forgotten. This led to the term the forgotten circulation. However, in the past decade, advances in lymphatic imaging and interventional techniques have now brought this circulatory system back into the limelight. In patients with congenital heart disease (CHD) especially single ventricle heart disease, the lymphatic system plays a key role in several often-devastating diseases and is a major cause of Fontan failure.

## Lymphatic Anatomy and Physiology

In general, lymphatic channels collect fluid from the peripheral organs and the peripheral tissue and transport centrally toward the main channel for lymphatic drainage called the thoracic duct. At the proximal end of the thoracic duct at the level of T11-L1, there is a sack called the cisterna chyli. This sack collects fluid from the lower extremities, the liver, and the mesentery. Under normal conditions, approximately 8 L of fluid a day are absorbed into the lymphatic ducts and approximately 2–3 L a day flow through the thoracic duct (1). The liver and the intestine each contribute approximately 40% of thoracic duct flow. The thoracic duct also receives lymphatic flow from the lungs and the heart. The thoracic duct then courses superiorly just anterior to the vertebral bodies and connects to the junction of the internal jugular vein and subclavian vein usually on the left side. Multiple studies using various lymphatic imaging modalities have demonstrated numerous thoracic duct anatomic variances (2, 3). A bicuspid valve sits at the mouth of the thoracic duct outlet and prevents reflux and blood back into the thoracic duct (4).

In the periphery terminal, lymphangions are made of a porous layer of lymphatic endothelial cells connected by microtubules to the extracellular matrix. When the tissue swells due to edema, the gap between lymphatic endothelial cells widens due to the connections to the extracellular matrix, allowing more fluid to enter the collecting lymphatic ducts. Once inside, the lymphatic ducts’ fluid is propelled forward by several forces. First, lymphatic ducts undergo rhythmic contractions due to a muscle layer in the duct wall (1, 5). Second, negative pressure exerted by the thorax during inspiration pulls fluid into the thoracic duct. Finally, it is also possible that muscle contractions contribute also to moving fluid forward, assuring that unidirectional flow are multiple valves throughout the system (5).

The Starling equation describes the rate of tissue and lymphatic fluid formation in relation to the difference between the oncotic and hydrostatic pressures (6). In addition, many extrinsic factors can also affect lymph flow and tissue fluid content. Medications, such as inotropes, have been shown to cause rhythmic lymphatic contractions in a dose-dependent manner (7). In patients with CHD, central venous pressure is particularly important as it increases lymphatic production, especially by the liver, and impedes lymphatic drainage at the lymphovenous junctions (8).

In addition, the patients with abnormal physiology with CHD likely have an underlying congenital or genetic susceptibility to developing lymphatic flow disorders. This is likely the reason why some patients with elevated CVP do not have lymphatic problems, while others with relatively low CVP can have severe lymphatic issues. Evidence for this has been published by Biko et al. who demonstrated nutmeg lung in fetuses with T2 imaging that is known to correlate with pulmonary lymphangiectasia (9). In most cases, the underlying genetics for these diseases is not known but in patients with certain syndromes, such as Noonan syndrome and trisomy 21, the genetic defects are known.

## Lymphatic Imaging

One of the most important advances in clinical lymphatics has been the development of minimally invasive lymphatic imaging techniques. Older imaging techniques, such as lymphoscintigraphy, are still used in some institutes as diagnostic tools and do have some benefits in patients with lymphatic flow disorders. However, to a large extent, magnetic resonance (MR) lymphangiography has now become the imaging modality of choice for most patients.

**FIGURE 1:**
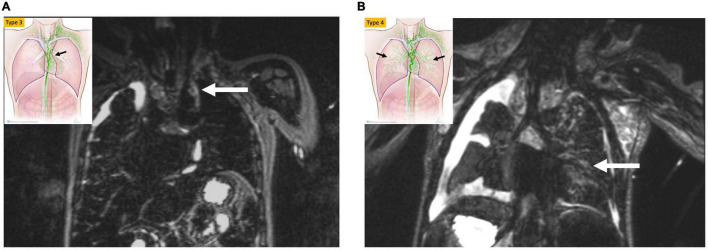
MIP coronal projection of T2 weighted lymphatic mapping showing patient with bright signal extending to the mediastinum (arrow) (type 3) in **(A)** and into the lung parenchyma (arrow) type 4 in **(B)**.

Non-contrast MR lymphangiography is one such technique that uses heavily weighted T2 sequences to image slow-moving non-bloody fluids (10). This technique is fast and non-invasive and has a good spatial resolution. However, it lacks dynamic information and is consequently insufficient for completely characterizing the lymphatic circulatory system. Recently, Biko et al. reported on the correlation between T2 findings in the thorax of patients who underwent superior cavopulmonary connection and Fontan outcomes (11). The paper demonstrated a strong correlation between poor Fontan outcomes and high-grade thoracic lymphatic abnormalities (Figure 1). This imaging should be used as a screening tool for all thoracic lymphatic abnormalities in all single ventricle patients prior to Fontan completion.

To fully characterize the circulatory system, anatomical, physiological, and flow information is needed. Dynamic contrast MR lymphangiography is now the standard method for imaging the central lymphatic system. In this technique, needles are placed inside lymph nodes or lymphatic ducts and the position is confirmed with a conventional iodinated contrast agent or by ultrasound. The needles are fixed and patients are transferred to an MRI scanner where they undergo dynamic and static contrast-enhanced MR imaging. This technique has a good spatial resolution as well as good temporal resolution allowing us to visualize the lymphatic flow and organ lymphatic perfusion. As a result, this imaging modality has transformed our understanding of lymphatic flow disorders and has allowed us to diagnose the etiology of several lymphatic diseases, such as protein-losing enteropathy (PLE) and plastic bronchitis (PB). Dori et al. published on the use of intranodal dynamic contrast lymphangiography (IN-DCMRL) in an animal model and then in a patient with PB (12, 13). Around the same time, Krishnamurthy et al. published on the use of this technique in patients with chylothorax (14). IN-DCMRL is the imaging modality of choice for viewing the lumbar lymphatic networks, cisterna chyli, and thoracic duct. However, abnormalities in flow originating from the two main contributors to thoracic duct flow, which are the liver and mesentery, are normally not visualized with this technique. Biko et al. recently published on the development of intrahepatic DCMRL (IH-DCMRL) (15). In a recent manuscript, Smith et al. showed the correlation between the different hepatic lymphatic flow patterns and systemic lymphatic diseases (16). In addition, Lemley et al. demonstrated that all patients with PLE have duodenal involvement with IH-DCMRL (17). In contrast, most patients without PLE do not have duodenal involvement with hepatic injection. Consequently, it is possible that intrahepatic imaging could be used as a screening tool for patients with PLE. The second major contributor to central lymphatic flow is the mesentery. Historically, this part of the lymphatic system has been difficult to access and has not been imaged in humans. However, this has now changed, and Dori et al. just published on the development of intramesenteric DCMRL (IM-DCMRL) (18). The utility of this imaging modality in patients with CHD is currently under investigation.

Ultrasound contrast lymphangiography is another technique that was recently published (19). This technique is good for assessing thoracic duct outlet patency which needs to be confirmed in all patients undergoing a thoracic duct decompression (TDD) procedure.

## Lymphatic Interventions

All patients with a suspected lymphatic abnormality should undergo cardiac catheterization to rule out a reversible cause of lymphatic failure, such as superior vena cava (SVC) stenosis, and to assess hemodynamics. In addition, medical management, including high-dose Aldactone, sildenafil, steroids, and other medications, should be optimized. Due to stroke risk, prior to lymphatic intervention, cardiac catheterization should be performed to determine all sources of the right to left shunting, including fenestration patency and veno-venous collaterals in patients with single ventricle anatomy and atrial septal defect (ASD) and ventricular septal defect (VSD) in other patients. In some cases, coil embolization of collateral vessels might be warranted as well as temporary balloon occlusion of shunts. However, because anticoagulation cannot be used during lymphatic procedures, careful consideration needs to be given to the decision to temporarily close a fenestration with a balloon and wire. The lymphatic patient population is particularly hypercoagulable, and systemic thrombus formation could also result in a thromboembolic stroke.

**FIGURE 2:**
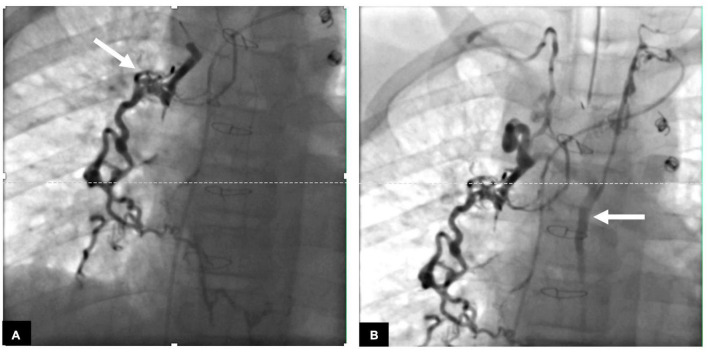
**(A)** AP fluoroscopy image of selective lymphatic embolization of a left sided dilated duct (arrow) with cyanoacrylate glue. **(B)** Contrast injection in the thoracic duct post-procedure demonstrating intact thoracic duct (arrow) with connections to both the left and right venous angles.

Lymphatic interventions in general can be divided into two groups, namely, those that are meant to occlude unwanted lymphatic ducts, such as thoracic duct embolization (TDE), and those that are meant to decompress the entire lymphatic system, such as lymphovenous anastomosis (LVA) (8). Minimally invasive procedures that occlude flow include ethiodized oil embolization, TDE, selective lymphatic duct embolization (SLDE), as well as several other procedures that are meant to selectively target lymphatic networks that are causing symptoms. In patients with CHD, selective embolizations that maintain thoracic duct patency are always preferable (Figure 2). Minimally invasive procedures to decompress the lymphatic system are now also available for all possible cardiovascular anatomies (20). Surgical procedures that occlude flow include surgical thoracic duct ligation (TDL) and pleurodesis. Most often, minimally invasive procedures have replaced the surgical occlusive procedures and are the interventional modality of choice, if available. Surgical procedures to decompress the thoracic duct include the Hraska procedure or innominate vein turndown (21–24). While the right to left shunting is of concern in both the minimally invasive and surgical decompression procedures, this often is not an issue and patients tolerate the procedures well. In some cases, banding of the internal jugular vein is needed (25).

## Lymphatic Flow Disorders

**FIGURE 3:**
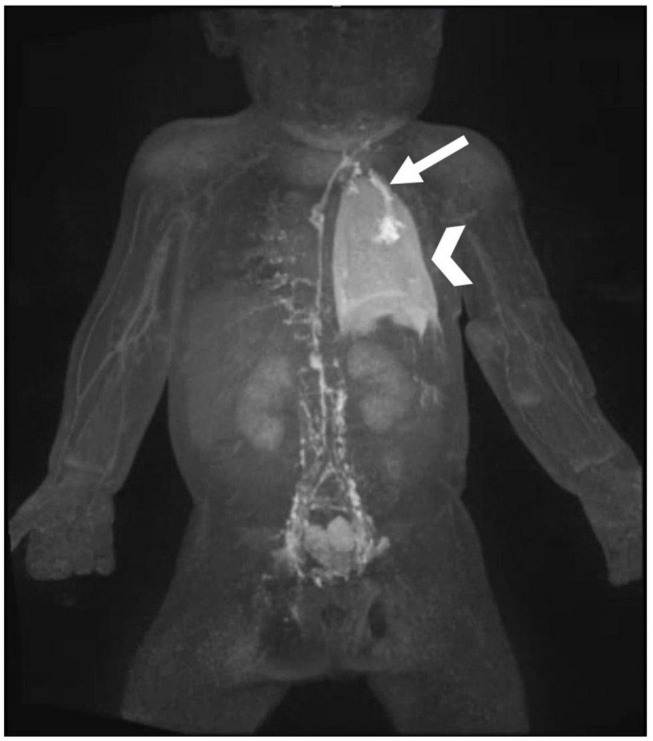
MIP coronal projection of IN-DCMRL demonstrating leak of contrast into the left pleural space (arrowhead) from dilated channel connected to the pericardium and originating from the distal TD (arrow).

Lymphatic flow disorders can lead to tissue edema, effusions, or organ dysfunction. Trauma, such as due to surgical procedure, can lead to an effusion. In patients with CHD, traumatic effusions are often associated with open-heart surgery. Historically traumatic leaks were thought to be due to injury to the thoracic duct. This is almost never the case, except during vascular ring repair, because the thoracic duct is posterior to the surgical field. Most often, traumatic effusions are due to injury to lymphatic ducts that are connected to the pericardium (Figure 3) (26). However, the most common causes of effusions, both in the chest and abdomen, are lymphatic production and conduction abnormalities which together with lymphatic channel dysfunction leads to abnormal organ perfusion and resultant leaks (26).

## Flow Disorders in the Thorax

**FIGURE 4:**
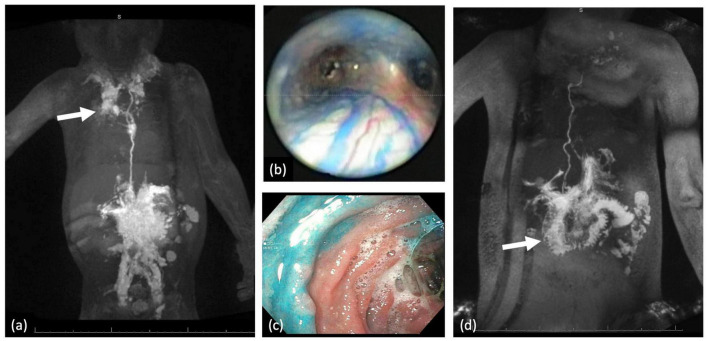
**(a)** MIP coronal projection of IN-DCMRL demonstrating mediastinal and bilateral PLPS more on the right (arrow). **(b)** In a patient with PB blue dye injection into the TD demonstrated dilated peribronchial lymphatic networks. **(c)** Blue dye injection into the liver demonstrating leak into the duodenal lumen. **(d)** MIP coronal projection of IH-DCMRL demonstrating leak into the duodenal lumen characteristic of PLE (arrow).

Three types of thoracic lymphatic flow abnormalities are often encountered in patients with CHD. These include pleural effusions, pericardial effusions, and PB (13, 26, 27). The etiology of all three abnormalities is almost always abnormal perfusion of the mediastinal, peribronchial networks, and pulmonary interstitial lymphatic networks which we have termed pulmonary lymphatic perfusion syndrome (PLPS) (Figures 4a,b). The degree of thoracic lymphatic abnormalities can be screened with a T2-weighted MRI which should be performed in all patients prior to Fontan completion.

## Pleural and Pericardial Effusions

**FIGURE 5:**
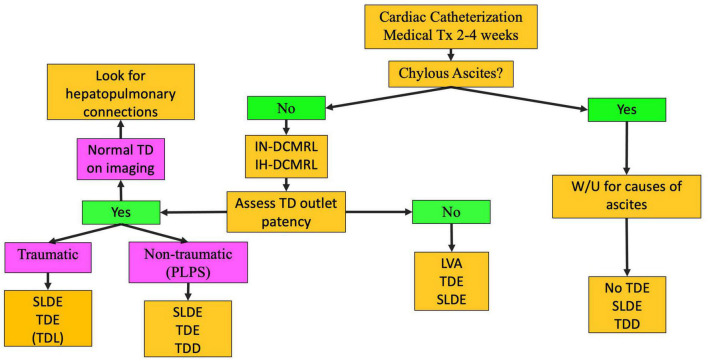
Chylothorax treatment pathway (TD-thoracic duct, DCMRL-dynamic contrast lymphangiography, IN-intranodal, IH-intrahepatic, SLDE-selective lymphatic duct embolization, TDL-thoracic duct ligation, TDE-thoracic duct embolization, TDD-thoracic duct decompression, LVA-lymphovenous anastomosis, PLPS-pulmonary lymphatic perfusion syndrome).

Pleural or pericardial effusions tend to occur mostly after Fontan completion but can be seen in neonates as well. In contrast, PB is most often encountered in single ventricle patients after Fontan palliation and is rare prior to this stage although it can also occur in patients with superior pulmonary connection depending on the severity of the lymphatic conduction abnormality. Chylothorax and chylopericardium are effusions that receive lymph draining from the mesentery and are diagnosed based on fluid triglyceride levels greater than 110 mg/dl if the patient is on a full-fat diet, high lymphocyte percentage (80–100%), and presence of chylomicrons. Our management protocol for these patients involves 2–4 weeks of conservative management with a low-fat diet or nothing be mouth (NPO) (Figure 5) (8). Cardiac catheterization and medical optimization are also performed. Octreotide can also be tried but in many cases is ineffective. If conservative management fails, patients undergo IN and IH-DCMRL to visualize the central conducting lymphatic networks as well as screen for the potential development of PLE. In single ventricle patients and in babies, SLDE is the first line of therapy. When this is not possible and ascites and PLE are not a concern, TDE or TDL is an option. Other procedures, such as pleurodesis, are rarely used in our institution and are almost always not needed.

## Plastic Bronchitis

**FIGURE 6:**
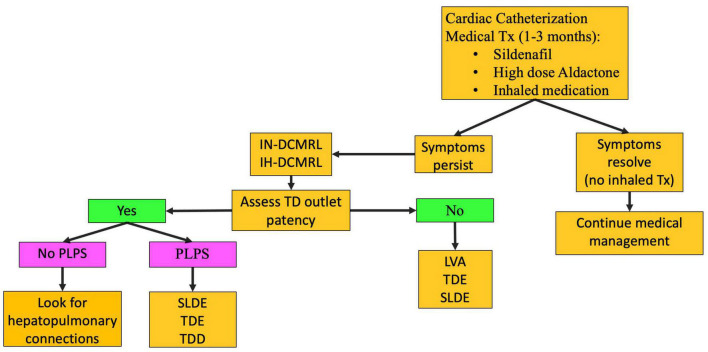
PB treatment pathway (TD-thoracic duct, DCMRL-dynamic contrast lymphangiography, IN-intranodal, IH-intrahepatic, SLDE-selective lymphatic duct embolization, TDL-thoracic duct ligation, TDE-thoracic duct embolization, TDD-thoracic duct decompression, LVA-lymphovenous anastomosis, PLPS-pulmonary lymphatic perfusion syndrome).

The PB occurs most often in patients with single ventricle CHD. However, it can occur in other settings. In the current era, the mortality of this disease is low with most patients living free of symptoms after a lymphatic intervention. This disease should be considered in any single ventricle patient presenting with a chronic cough or diagnosis of asthma not responsive to medication. The etiology of PB is retrograde perfusion of the peribronchial lymphatic networks leading to inflammation and edema of the airway mucosa (13, 27). When the barrier for protein leak is overcome, lymphatic fluid containing proteinaceous material is instilled into the airway. Proteinaceous material, including fibrinogen, becomes sticky in contact with a rare-forming gel-like material that can fill the airway forming the cast. Larger casts can put the patient at risk for respiratory failure from asphyxiation. Conservative management of PB involves inhaled bronchodilators, inhaled steroids, and pulmonary vasodilators (Figure 6). Cardiac catheterization is performed as well. If symptoms persist, medications aimed at breaking down the casts are used, including inhaled tissue plasminogen activator. However, if fibrinolytics are needed to improve symptoms, there is active lymph leaking into the airway and lymphatic imaging and intervention are needed. After IN and IH-DCMRL, SLDE is the first line of treatment.

If pleural effusion or PB symptoms persist despite lymphatic intervention, then TDD or orthotopic heart transplant (OHT) should be considered. In addition, it is reasonable to consider surgical TDD as the fenestration technique during the Fontan procedure in patients with high-grade thoracic lymphatic abnormalities as determined by T2 imaging.

**FIGURE 7:**
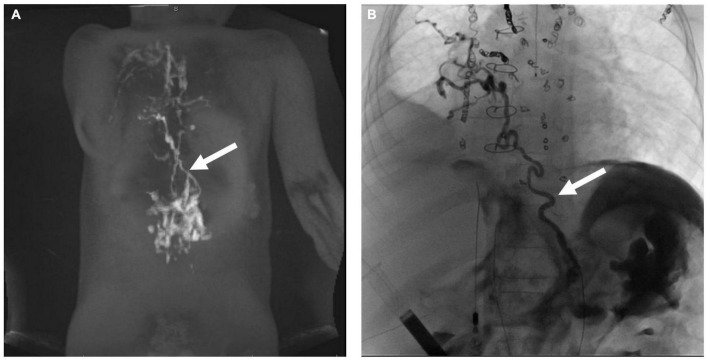
**(A)** MIP coronal projection of IH-DCMRL demonstrating hepatopulmonary connection (arrow). **(B)** AP fluoroscopic image showing the hepatopulmonary connection after selective embolization (arrow).

Irrespective of clinical presentation in patients with thoracic lymphatic flow disorders, if central lymphatic imaging demonstrates a normal thoracic duct flow pattern without perfusion of the mediastinum or the lungs, then TDE or TDL should not be performed as it can lead to worsening of symptoms. In these cases, the etiology is most often hepatopulmonary connections that bypass the central lymphatic conduction system, including the thoracic duct (Figure 7) (16). Selective embolization of hepatopulmonary connections will lead to a resolution of symptoms in most cases while maintaining central lymphatic flow.

## Lymphatic Abnormalities in the Abdomen

### Protein-Losing Enteropathy

The PLE is the most commonly encountered lymphatic flow abnormality in the abdomen, especially in patients with single ventricle physiology. PLE is defined as the loss of albumin and other proteins in the intestinal tract. This is a potentially life-threatening disease affecting approximately 5–15% of single ventricle patients (28, 29). Patients present with low albumin and other common symptoms include diarrhea, edema, ascites, weight loss, and malabsorption. Diagnosis can often be confirmed with a stool alpha-1 antitrypsin. The etiology of PLE in all patients with single ventricle physiology is abnormal perfusion of the duodenum by increased liver lymphatic production and, in some cases, increased mesenteric production as well (30). This leads to duodenal wall edema, inflammation, and lymphangiectasia. When the barrier to protein leak is broken, lymphatic fluid will leak into the duodenal lumen leading to symptoms. In the later stages of the disease, endoscopy has demonstrated holes formed in the duodenal wall (Figure 4c).

**FIGURE 8:**
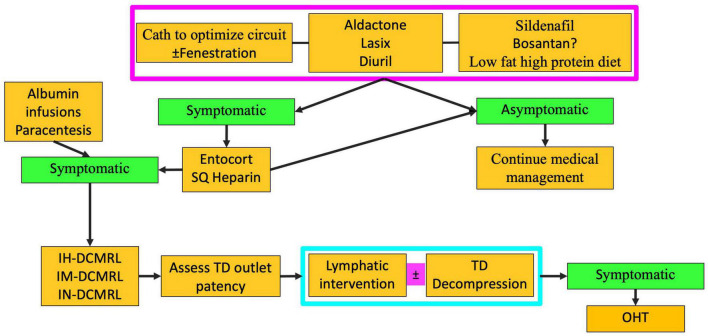
Algorithm for PLE treatment (TD-thoracic duct, DCMRL-dynamic contrast lymphangiography, IN-intranodal, IH-intrahepatic, IM-intramesenteric, SQ-subcutaneous, OHT-orthotopic heart transplant).

**FIGURE 9:**
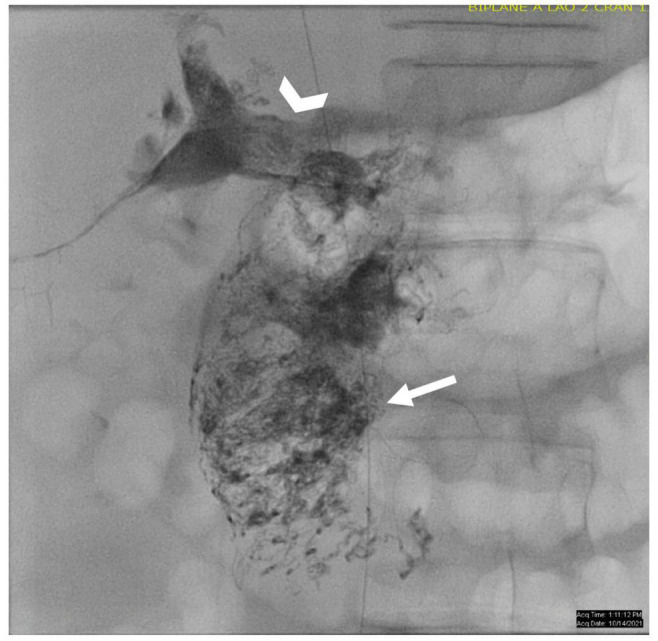
AP fluoroscopic image of the duodenum after periduodenal (arrow) and hepatoduodenal (arrowhead) lymphatic embolization.

Conservative management of PLE involves diuretics, including high-dose Aldactone and sildenafil (Figure 8) (8). The role of a low-fat high-protein diet is not clear but has also been tried. Cardiac catheterization must be performed to determine hemodynamics and any reversible Fontan pathway obstruction. Fenestration creation or recreation can also be attempted. Those who are asymptomatic with conservative management are continued to be monitored. However, those that remain symptomatic are then started on enteric steroids. If steroids fail to control symptoms, lymphatic imaging with IN and IH-DCMRL and intervention are warranted (Figure 4d). Interventions for PLE include embolization of hepatoduodenal and periduodenal lymphatic networks and procedures to lower pressure in the lymphatic system, such as TDD (Figure 9). When treatments fail Fontan takedown, VAD placement or OHT is considered.

### Ascites

Chylous ascites can occur as a result of traumatic lymphatic leaks. However, most often in patients with CHD, ascites are non-chylous as a result of mesenteric lymphatic congestion. Paracentesis can be helpful for diagnostic purposes and to relieve symptoms. Imaging with DCMRL in patients with chylous ascites can help localize the leak which then can be glue embolized. In patients with non-chylous ascites, TDD should be considered.

### Multicompartment Lymphatic Failure

**FIGURE 10:**
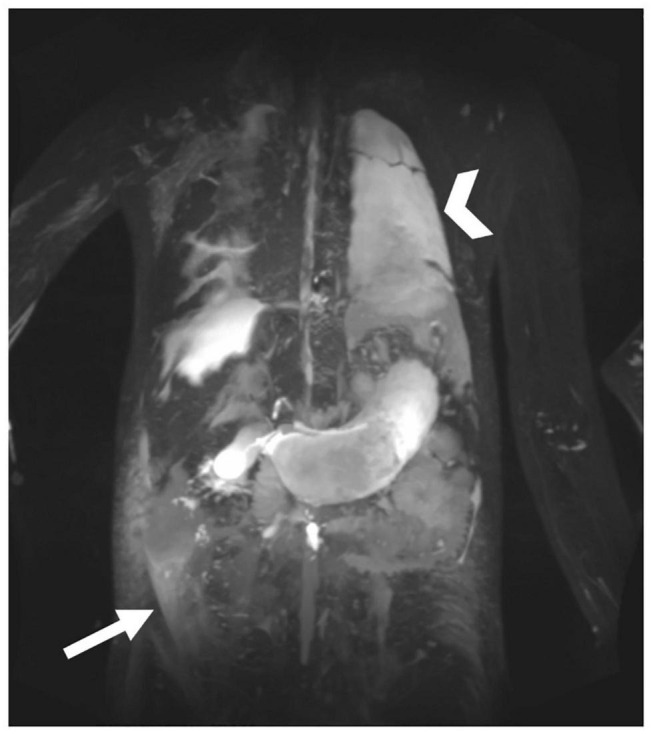
MIP coronal projection of T2 imaging in patient with multicompartment lymphatic failure including ascites (arrow), PLE, edema, and chylothorax (arrowhead).

**FIGURE 11:**
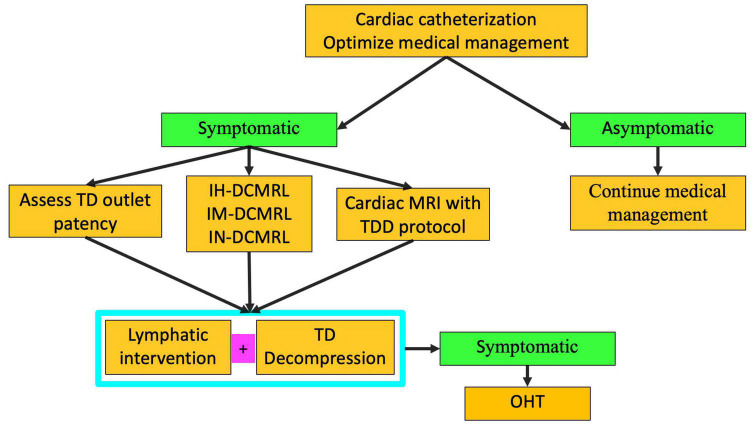
Algorithm for multicompartment failure (TD-thoracic duct, DCMRL-dynamic contrast lymphangiography, IN-intranodal, IH-intrahepatic, IM-intramesenteric, TDD-thoracic duct decompression, OHT-orthotopic heart transplant).

Multicompartment lymphatic failure is defined as lymphatic perfusion abnormality involving at least two compartments, including thorax, abdomen, and soft tissue (Figure 10) (8). In both neonates and older patients, this is a difficult condition to manage. Irrespective of age, TDE or TDL should never be performed in these patients. Multicompartment lymphatic imaging should be performed in all cases (Figure 11). Selective embolization procedures in some cases can be performed but careful consideration should be paid to the consequences that these procedures can have on the other compartments. Normally, a combination of conservative management and TDD procedures, such as LVA, can resolve symptoms in neonates. In adults, selective embolization procedures and TDD can also lead to a resolution of symptoms. If, however, symptoms persist in most cases, OHT or assist device is needed.

## Conclusion

Patients with CHD especially after single ventricle palliation are susceptible to a variety of lymphatic abnormalities of both the thorax and the abdomen. Imaging is the key step in the diagnosis of the ideology as well as for planning interventions. Imaging should be used to screen all patients with single ventricle physiology prior to undergoing Fontan palliation. Selective lymphatic interventions are the procedures of choice when possible.

## Author Contributions

Both authors listed have made a substantial, direct, and intellectual contribution to the work, and approved it for publication.

## Conflict of Interest

The authors declare that the research was conducted in the absence of any commercial or financial relationships that could be construed as a potential conflict of interest.

## Publisher’s Note

All claims expressed in this article are solely those of the authors and do not necessarily represent those of their affiliated organizations, or those of the publisher, the editors and the reviewers. Any product that may be evaluated in this article, or claim that may be made by its manufacturer, is not guaranteed or endorsed by the publisher.
